# Factors associated with the risk of gingival disease in patients with rheumatoid arthritis

**DOI:** 10.1371/journal.pone.0186346

**Published:** 2017-10-12

**Authors:** Liang-Gie Huang, Gin Chen, Der-Yuan Chen, Hsin-Hua Chen

**Affiliations:** 1 Department of Stomatology, Taichung Veterans General Hospital, Taichung, Taiwan; 2 School of Dentistry, National Yang-Ming University, Taipei, Taiwan; 3 School of Dentistry, Chung-Shan Medical University, Taichung, Taiwan; 4 Department of Medical Education, Taichung Veterans General Hospital, Taichung, Taiwan; 5 School of Medicine, National Yang-Ming University, Taipei, Taiwan; 6 Division of Allergy, Immunology and Rheumatology, Department of Internal Medicine, Taichung Veterans General Hospital, Taichung, Taiwan; 7 Institute of Biomedical Science and Rong Hsing Research Center for Translational Medicine, Chung-Hsing University, Taichung, Taiwan; 8 School of Medicine, Chung-Shan Medical University, Taichung, Taiwan; 9 Department of Medical Research, Taichung Veterans General Hospital, Taichung, Taiwan; 10 Institute of Public Health and Community Medicine Research Center, National Yang-Ming University, Taipei, Taiwan; The Ohio State University, UNITED STATES

## Abstract

Gingival disase and rheumatoid arthritis (RA) are linked at both the epidemiologic and pathogenesis levels. In this study, we aimed to identify environmental factors associated with RA and gingival disease and to investigate factors that protect the gingival tissue in RA patients. This retrospective study analyzed 754 RA patients with gingival disease selected from the NHANES database who completed the mobile examination center interview/examination between 1999 and 2004. Data collected included demographics, lifestyle, dietary intake, and biomarkers. The study included 173 RA patients with gingival disease. Multivariate logistic regression analysis showed that the odds of gingival disease were significantly increased with male gender. However, the odds of gingival disease was significantly decreased with increased vitamin C intake (OR = 0.996, p = 0.041), and higher serum vitamin D levels (OR = 0.979, p = 0.011). Given the significant association between the prevalence of gingival disease and RA, identification of risk factors of gingival disease will be useful as a screening tool in national health surveys to improve the management of periodontal disease in patients with RA.

## Introduction

Rheumatoid arthritis (RA) is an autoimmune disease in which chronic synovial inflammation resulting from impaired immune homeostasis leads to joint destruction and bone erosion [1, 2]. Although, it has been suggested that RA can be triggered in genetically predisposed subjects by an inadequate immune response to environmental challenges such as bacteria and viruses [3], the pathogenesis of the disease is not well understood. Factors that play an important role in RA pathogenesis include tumor necrosis factor (TNF)-α, IL-6, autoreactive Th17 cells producing IL-17, and regulatory T cells (Tregs) [4–6]. Traditional therapy for RA includes the use of synthetic disease-modifying anti-rheumatic drugs (DMARDs) to limit inflammation and slow down structural changes to the joints [7]. However, based on the role played by the immune system in RA, novel therapeutic regimens are also used and include propagation of Tregs, use of biologic DMARDs targeting IL-17, IL-6, and granulocyte-macrophage colony-stimulating factor, TNF inhibitors, anti-CD20, and immunoproteasome inhibitors [8].

Gingival disease, which is triggered by oral microbial etiologic factors, has an immunoinflammatory pathogenesis where dysregulation of the inflammatory and immune pathways results in chronic inflammation, and a breakdown of the supporting structures including alveolar bone and periodontal ligaments that surround the teeth [9, 10]. Based on findings that the pathogenesis of RA and gingival disease could be driven by a similar profile of inflammatory cells and pro-inflammatory cytokines, it has been suggested that there could be a non-causal relationship between the two diseases [11]. *Porphyromonas gingivalis*, the major oral pathogen associated with gingival disease, expresses peptidyl arginine deiminase (PAD) which catalyzes the citrullination of arginine [12]. Citrullinated antigens have been demonstrated in the periodontium of gingival disease patients [12], and circulating antibodies to *P*. *gingivalis* are correlated with the presence of anti-citrullinated protein antibodies (ACPA) [13]. Autoimmunity in RA is characterized by the presence of antibodies to human citrullinated α-enolase, which cross-react with citrullinated enolase from *P*. *gingivalis* [14].

A number of studies have reported that the prevalence of gingival disease in subjects with RA was significantly higher compared with the general population; although, it is not clear if the clinical course of gingival disease is more severe in these subjects [15–18]. However, other data showed no significant correlation in the prevalence of gingival disease between RA patients and controls [16, 19, 20]. These inconsistencies are thought to be due to differences in sample sizes, demographics, disease criteria, and in adjustments for confounding factors between studies [16]. Interestingly, mechanical periodontal treatment, such as ultrasonic scaling and root planning for gingival disease, also attenuated the severity of RA [21].

The National Health and Nutritional Examination Survey (NHANES) provides national estimates on selected health characteristics in the United States over 2-year periods. NHANES uses a stratified multistage probability sampling strategy where non-institutionalized civilian individuals are selected, interviewed, and undergo physical examinations in mobile examination centers. Data from NHANES is organized by subject matter. Since the data files comprising NHANES include a number of demographic and socioeconomic variables including age, gender, race, ethnicity, income, education, and marital status, the analysis of subjects from NHANES is reliable, representative and multidimensional [22]. NHANES data collected between 2009–2012 showed that 46% of adults in the US had periodontitis, and that there were significant disparities in the burden of periodontitis between different socio-demographic groups [23].

Since RA and gingival diseases are thought to be related at the epidemiological level, it is important to identify environmental/genetic factors associated with these diseases in order to understand mechanisms underlying disease pathogenesis. It is also important to investigate factors that protect against the development of periodontal disease in RA patients, which could help in the adoption of appropriate public health measures for oral disease prevention [24]. In this study, we aimed to assess the association of periodontal disease with RA, and used the NHANES database to investigate risk factors of gingival disease in RA patients.

## Methods

### Data source

Data for this study were obtained from the Centers for Disease Control and Prevention (CDC), National Center for Health Statistics (NCHS), National Health and Nutrition Examination Survey Data (NHANES). Hyattsville, MD: U.S. Department of Health and Human Services, Centers for Disease Control and Prevention, 1999–2004. Further information about background, design and operation are available on the NHANES website (http://wwwn.cdc.gov/nchs/nhanes). All NHANES data are de-identified and analysis of the data does not require informed consent. This study was approved by the Institutional Review Board of Taichung Veterans General Hospital Taiwan (IRB number: CE16280A). Simultaneous data for dental examinations as well as RA were only available for the period between 1999 and 2004.

### Study population

**Fig 1:**
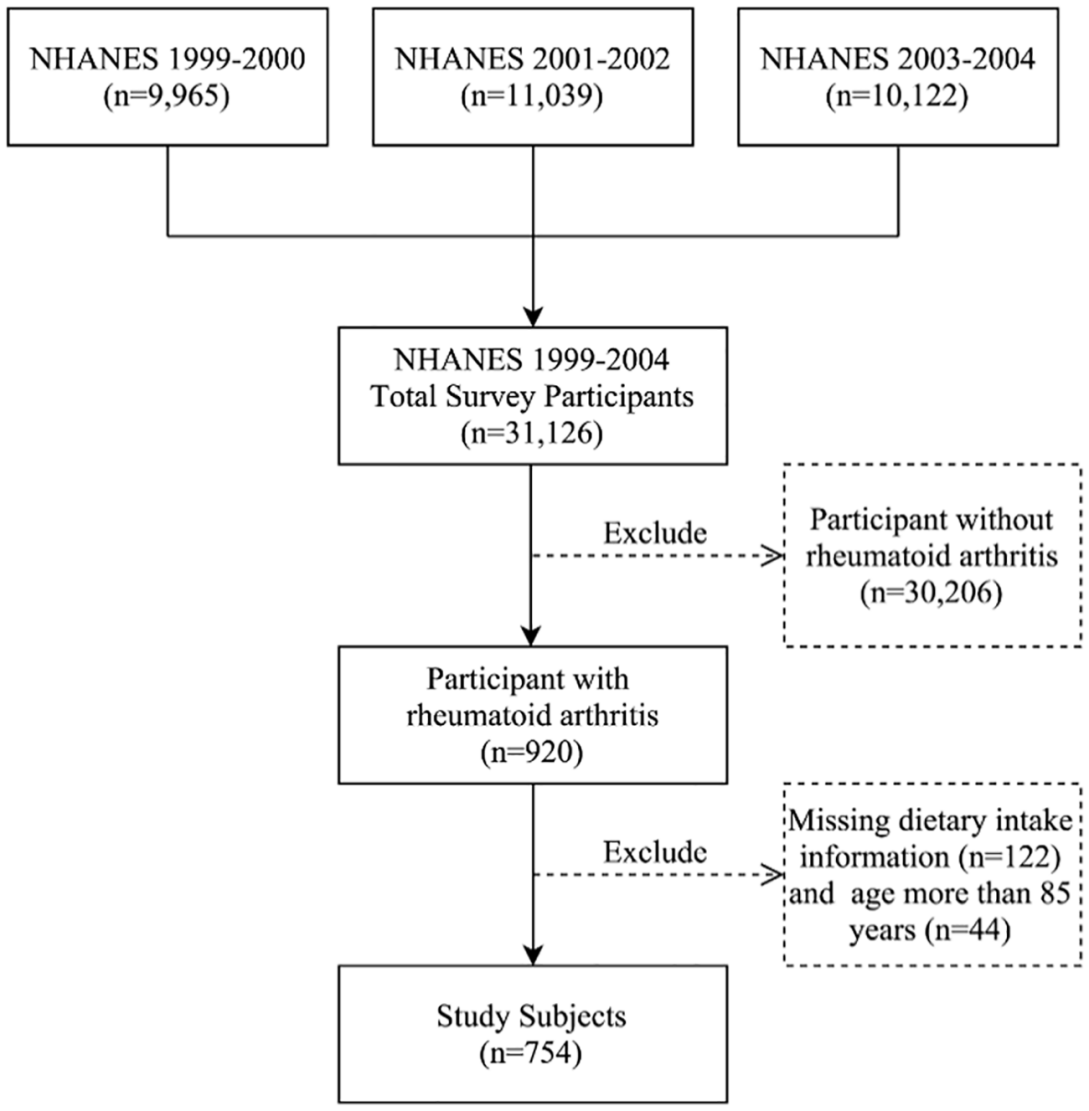
Flow chart for study subject selection.

This retrospective study evaluated a total of 31,126 participants selected from the NHANES database who completed the mobile examination center interview/examination between 1999 and 2004. The final analysis included patients who self-reported a diagnosis of rheumatoid arthritis which was given by a doctor or health professional. Since dietary/nutrition status was an important focus of this study, subjects with missing dietary data were excluded. Subjects >85 years of age were also excluded because age in years was top coded at aged 85 years (ie, specific ages of participants about 85 years of age was not reported) in NHANES to reduce the risk of disclosure. A total of 754 participants with rheumatoid arthritis were included in the final analysis. A flow diagram of the selection process is shown in Fig 1.

### Study variables

#### Gingival disease (periodontitis)

Oral health examinations were performed by trained dentists who held a state dental license in a U.S. jurisdiction. All examinations were conducted in mobile examination centers. Measurements were made at six sextants per tooth, and the periodontal examination assessed the loss of gingival attachment and bleeding upon probing (BOP). The results of oral health examination were then recorded in the Oral Health-Recommendation of Care file. Detailed descriptions were provided in the dental examiners’ procedures manual, available at: https://www.cdc.gov/nchs/data/nhanes/oh-e.pdf. The “periodontal needs” variable determined whether the subject suffered from gingival disease (periodontitis). For periodontal assessment (both upper and lower) the following were evaluated: periodontal pockets, recession, loss of attachment, and bleeding for the maxillary sites assessed. Detailed description of the oral health examination can be found at: https://wwwn.cdc.gov/Nchs/Nhanes/2001-2002/OHXREF_B.htm#OHAROCGP

#### Demographics

Age, sex, and race/ethnicity were obtained from the NHANES database. The duration of arthritis was calculated as follows: age at screening minus age at which patient received a diagnosis of arthritis.

#### Behavioral data

Cigarette smoking status was recorded. Alcohol consumption was classified into three categories: 1) current alcohol drinkers were those who consumed at least 12 drinks in their entire life, and had consumed alcohol on at least one day in the past 12 months; 2) former drinkers were those who had consumed at least 12 drinks during their lifetime, but had not consumed alcohol in the past 12 months; 3) never drinkers were those who reported never having consumed at least 12 drinks during their lifetime. Determination of routine dental checkups were ascertained by the question “During the past 3 years, have you been to the dentist for routine check-ups or dental cleanings?” Physical activity patterns were defined by responses to the question “Which of these four sentences best describes your usual daily activities?” Participants who were sedentary were classified in the “mainly sit” category. Participants who walked a lot, or carried light or heavy loads were classified in the “not mainly sit” category.

#### Diet/Nutrition data

Information on dietary intake was collected by asking the study participants to recall and record all foods and beverages consumed during the 24-hour period prior to the interview (midnight to midnight). The information was imported into the University of Texas Food Intake Analysis System (FIAS), and dietary nutrient intake was calculated by the US Department of Agriculture (USDA) 1994–98 Survey Nutrient Database. Detailed descriptions of the dietary interview methods are provided in the NHANES Dietary Interviewer’s Training Manual, available at: http://www.cdc.gov/nchs/nhanes/nhanes1999-2000/current_nhanes_99_00.htm. The Total Nutrient Intakes files included daily sodium intake, Vitamin C intake, alpha-carotene intake, beta-cryptoxanthin intake, and Vitamin E intake.

#### Biological data

Serum 25-hydroxyvitamin D (nmol/L) levels were measured at the National Center for Environmental Health of the CDC, Atlanta, GA using the DiaSorin RIA kit (Stillwater MN) in NHANES 2001–2006. However, later surveys used the liquid chromatography-tandem mass spectrometry (LC-MS/MS) method. The CDC recommends using the total 25(OH) D in SI units (nmol/L) measured directly by LC-MS/MS or the predicted LC-MS/MS equivalent. In the present study, we used the LC-MS/MS-equivalent data [25]. In NHANES, total bilirubin levels are determined using a Beckman Synchron LX20. C-reactive protein (CRP) is quantified by latex-enhanced nephelometry using a Behring nephelometer, and serum concentrations of vitamins E, Alpha-carotene, Beta-cryptoxanthin, lutein/zeaxanthin, and trans-lycopene are measured using high performance liquid chromatography with photodiode array detection. Detailed descriptions of blood collection and processing procedures are provided in the NHANES Laboratory/Medical Technologists Procedures Manual or NHANES website [26].

#### Biomarker variables

According to NHANES 2003–2004 data documentation and analytic notes, Vitamins A, E and Carotenoids were analyzed using a comparable HPLC method at Craft Technologies, Inc. (CTI) in 2003–2004 (see https://wwwn.cdc.gov/nchs/nhanes/search/datapage.aspx?Component). Since crossover studies between CDC/NCEH and CTI which were done in early 2003 and late 2004 exhibited differences between the methods for some analytes, in NHANES 2003–2004, the data for vitamin E and carotenoids were adjusted using Deming regression.

### Statistical analysis

Continuous variables were expressed as mean ± standard error, and categorical variables as unweighted counts (weighted %). Differences in categorical variables were determined by the Chi-square test, and differences in continuous variables were determined using the Complex Samples General Linear Model (CSGLM).

Univariate and multivariate logistic regression analyses were performed to identify protective factors significantly associated with the risk of gingival disease. Significant variables (p value < 0.05) identified in univariate logistic regression analyses were selected and evaluated by multivariate logistic regression model, with multivariate models simultaneously adjusted for age, gender, smoking, physical activity, vitamin C intake, serum vitamin D, serum Beta-cryptoxanthin and serum vitamin E.

All analyses included special sample weights [2/3 * WTDR4YR (Dietary day one 4-year sample Weight) for 1999–2002; 1/3 * WTDRD1 (Dietary day one 2-year sample weight) for 2003–2004], stratum, and primary sampling units (PSU) per recommendations from NCHS, to address oversampling, non-response, non-coverage, and to provide nationally representative estimates [27]. All statistical assessments were two sided and evaluated at the 0.05 level of significance. Statistical analyses were performed by IBM SPSS statistical software version 22 for Windows (IBM Corp., Armonk, New York, USA).

## Results

Of the 31,126 participants selected from the NHANES database who completed the mobile examination center interview/examination between 1999 and 2004, a total of 920 participants with rheumatoid arthritis were included in this study. Subjects who had no dietary intake information (n = 122), and subjects aged > 85 years old (n = 44) were excluded, and a total of 754 participants were included in the final analysis (Fig 1). The study population comprised 452 females (59%) and 302 males (41%), and most subjects was ≤ 60 years of age (60.7%). Using NHANES dietary sample weight, the analytic sample size was equivalent to a population-based sample size of 9,489,823 participants.

### Subject characteristics

Among the arthritic patients, there were 173 (22.94%) patients with gingival disease. Demographics and dietary characteristics of the study participants are summarized in Table 1. Compared with patients with gingival disease, a significantly higher percentage of patients with no gingival disease were older subjects (42.4% vs. 28.1%, p = 0.023), female (61.1% vs. 51.1%, p = 0.044), non-smokers (73.3% vs. 57.5%, p = 0.003) and classified as “not mainly sit” (69.9% vs. 54.7%, p = 0.017). Analysis of dietary characteristics showed that patients with no gingival disease had a significantly higher mean vitamin C and Beta-cryptoxanthin intake compared with patients with gingival disease (98.82 vs. 68.37 mg, p = 0.008; 152.33 vs. 91.15 mcg, p = 0.024, respectively). Analysis of biomarkers showed that patients without gingival disease had a significantly higher mean levels of serum vitamin D, alpha-carotene, beta-cryptoxanthin and trans-lycopene compared with patients with gingival disease (60.32 vs. 52.48 nmol/L, p = 0.019; 3.14 vs. 2.07 mg/dL, p = 0.002; 8.56 vs. 6.39 ug/dL, p = 0.003; 21.22 vs. 18.16 ug/dL, p = 0.045, respectively).

**Table 1:** Demographics and dietary characteristics of study participants (Unweighted n = 754, Weighted N = 9,489,823).

Variables	Total (n = 754)^a^	Without Gingival disease (n = 581)^a^	With Gingival disease (n = 173)^a^	p-value
**Demographic**				
Age				0.023*
≤ 60 years old	329 (60.7)	238 (57.6)	91 (71.9)	
> 60 years old	425 (39.3)	343 (42.4)	82 (28.1)	
Gender				0.044*
Female	452 (59.0)	362 (61.1)	90 (51.1)	
Male	302 (41.0)	219 (38.9)	83 (48.9)	
Race				0.159
Non-Hispanic White	339 (67.5)	279 (69.7)	60 (59.3)	
Non-Hispanic Black	207 (17.3)	145 (15.4)	62 (24.5)	
Mexican American	157 (4.5)	116 (4.1)	41 (5.7)	
Other Hispanic	26 (5.1)	22 (5.5)	4 (3.9)	
Other Race	25 (5.6)	19 (5.3)	6 (6.5)	
Length of arthritis (years)	13.78±0.60	13.80±0.65	13.70±1.45	0.945
**Health behavior**				
Smoking				0.003*
Yes	184 (30.1)	122 (26.7)	62 (42.5)	
No	570 (69.9)	459 (73.3)	111 (57.5)	
Alcohol				0.847
Never	126 (15.6)	90 (15.1)	36 (17.3)	
Former	223 (27.2)	179 (27.1)	44 (27.7)	
Current	375 (57.2)	285 (57.9)	90 (55.0)	
Routine dental checkups				0.655
No	90 (49.6)	65 (48.2)	25 (53.9)	
Yes	74 (50.4)	57 (51.8)	17 (46.1)	
Physical activity				0.017*
Mainly sit	259 (33.4)	187 (30.1)	72 (45.3)	
Not mainly sit	492 (66.6)	391 (69.9)	101 (54.7)	
**Diet**				
Daily sodium intake (mg)	3106.79±78.86	3132.13±89.50	3013.77±173.60	0.554
Vitamin C intake (mg)	92.30±6.77	98.82±8.51	68.37±6.32	0.008*
Alpha-carotene intake (mcg)	351.72±63.61	358.32±67.19	326.16±148.94	0.841
Beta-cryptoxanthin intake (mcg)	139.77±13.99	152.33±15.28	91.15±22.45	0.024*
Vitamin E intake (mg)	6.16±0.24	6.15±0.28	6.22±0.44	0.900
**Biomarker**				
Serum Vitamin D (nmol/L)	58.74±1.51	60.32±1.74	52.48±2.59	0.019*
Bilirubin (mg/dL)	0.65±0.01	0.65±0.02	0.64±0.03	0.834
C-reactive protein (mg/dL)	0.61±0.03	0.58±0.04	0.71±0.07	0.103
Serum Alpha-carotene (ug/dL)	2.92±0.19	3.14±0.22	2.07±0.26	0.002*
Serum Beta-cryptoxanthin (ug/dL)	8.12±0.48	8.56±0.56	6.39±0.53	0.003*
Lutein (mIU/mL)	13.92±0.44	14.06±0.48	13.39±0.79	0.445
Trans-lycopene (ug/dL)	20.60±0.71	21.22±0.77	18.16±1.30	0.045*
Serum Vitamin E (ug/dL)	258.91±11.43	248.92±11.40	294.98±24.07	0.063

*Note*. Continuous variables were shown mean ± standard error; categorical variables were shown unweighted counts (weighted %).

* Significant difference between with and without gingival disease, p < 0.05.

^a^ Numbers may not add to full sample due to missing data.

### Logistic regression analysis of protective factors associated with the risk of gingival disease

The result of univariate logistic regression analysis revealed that the odds of gingival disease was significantly decreased in subjects >60 years of age (OR = 0.530, p = 0.024), increased vitamin C intake (OR = 0.997, p = 0.023), increased serum vitamin D (OR = 0.984, p = 0.018), and increased serum beta-cryptoxanthin (OR = 0.943, p = 0.015). In addition, non-smoking participant (OR = 0.493, p = 0.004) and not mainly sit participant (OR = 0.521, p = 0.018) were associated with decreased odds of gingival disease. However, the odds of gingival disease were significantly increased in males (OR = 1.506, p = 0.045), Non-Hispanic Black (OR = 1.874, p = 0.025) and increased serum vitamin E levels (OR = 1.002, p = 0.046). The tests of model found the effects for race were not significant (p = 0.162); hence, race was not selected into the multivariate regression model. (Table 2).

**Table 2:** Logistic regression analysis of protective factors associated with the risk of gingival disease.

Variables	Univariate		Multivariate	
	Odds Ratio(95%CI)	p-value	Odds Ratio(95%CI)	p-value
**Demographic**				
Age				
≤ 60 years old	Reference		Reference	
> 60 years old	0.530(0.306,0.918)	0.024*	0.575(0.244,1.357)	0.198
Gender				
Female	Reference		Reference	
Male	1.506(1.009,2.248)	0.045*	2.225(1.049,4.716)	0.038*
Race				
Non-Hispanic White	Reference			
Non-Hispanic Black	1.874(1.086,3.234)	0.025*		
Mexican American	1.615(0.906,2.880)	0.102		
Other Hispanic	0.848(0.237,3.032)	0.795		
Other Race	1.427(0.544,3.739)	0.461		
Length of arthritis (years)	0.999(0.981,1.018)	0.946		
**Health behavior**				
Smoking				
Yes	Reference		Reference	
No	0.493(0.310,0.786)	0.004*	0.668(0.327,1.367)	0.259
Alcohol drinking				
Never	Reference			
Former	0.889(0.449,1.762)	0.730		
Current	0.827(0.402,1.702)	0.598		
Routine dental checkups				
No	Reference			
Yes	0.797(0.281,2.256)	0.656		
Physical activity				
Mainly sit	Reference		Reference	
Not mainly sit	0.521(0.305,0.890)	0.018*	0.442(0.194,1.010)	0.053
**Diet**				
Daily sodium intake(mg)	1.000(0.999,1.000)	0.571		
Vitamin C intake(mg)	0.997(0.994,0.999)	0.023*	0.996(0.992,0.999)	0.041*
Alpha-carotene intake(mcg)	1.000(0.999,1.000)	0.856		
Beta-cryptoxanthin intake(mcg)	0.999(0.997,1.000)	0.135		
Vitamin E intake(mg)	1.003(0.949,1.061)	0.900		
**Biomarker**				
Serum Vitamin D (nmol/L)	0.984(0.971,0.997)	0.018*	0.979(0.964,0.995)	0.011*
Bilirubin (mg/dL)	0.905(0.341,2.399)	0.837		
C-reactive protein (mg/dL)	1.138(0.972,1.334)	0.106		
Serum Alpha-carotene (ug/dL)	0.781(0.608,1.004)	0.053		
Serum Beta-cryptoxanthin (ug/dL)	0.943(0.900,0.988)	0.015*	0.980(0.944,1.018)	0.278
Lutein (mIU/mL)	0.988(0.954,1.022)	0.456		
Trans-lycopene (ug/dL)	0.972(0.945,1.000)	0.053		
Serum Vitamin E (ug/dL)	1.002(1.000,1.003)	0.046*	1.001(0.999,1.004)	0.355

*Note*. Multivariate regression models simultaneously adjusted for age, gender, smoking, physical activity, vitamin C intake, serum vitamin D, serum Beta-cryptoxanthin and serum vitamin E.

* Significant factor, p < 0.05.

Multivariate logistic regression analysis found that the odds of gingival disease were significantly increased in males (OR = 2.501, p = 0.02). However, the odds of gingival disease was significantly decreased with higher vitamin C intake (OR = 0.996, p = 0.041), and 2) serum vitamin D levels (OR = 0.979, p = 0.011).

## Discussion

In this study, we showed that the odds of gingival disease were significantly increased in males (>60 and <85 years of age) with RA. Older patients with RA had a lower association of gingival disease. Additionally, our data suggested that increased vitamin C intake or vitamin D serum levels were associated with a decrease in the incidence of adult periodontitis in RA patients.

The association between gingival disease and RA has been explained by two different hypotheses. According to the “two hit” model, a primary”hit” of gingival disease induces chronic inflammation which is followed by a secondary arthritogenic “hit” resulting in an increase in mediators of inflammation such as C-reactive protein, cytokines, prostanoids, and TNFα [28]. The second model is based on the activation of deamination enzymes by *P*. *gingivalis*, leading to citrullination of proteins and production of anticyclic citrullinated peptide autoantibodies which play a role in development of RA [29]. Studies investigating the link between gingival disease and RA reported a significantly higher prevalence of gingival disease among RA patients compared with osteoarthritic controls [12]. In a systematic review of 26 studies, 24 studies showed a significant association between gingival disease and RA [21]. Additionally, a systematic review of 19 studies showed a positive outcome of gingival disease treatment on the clinical features of RA [18].

Important risk factors for gingival disease include 1) demographic factors such as age, race and gender, 2) genetic factors, 3) lifestyle factors such as smoking, and alcohol consumption, and 4) diseases such as diabetes mellitus, obesity, and metabolic syndrome [23, 30, 31]. Specific genetic markers for gingival disease have not been identified but genetic predisposition is thought important for both the onset and progression of the disease [30]. Although smoking is the major risk factor for RA as well as gingival disease, it is a risk factor only for ACPA- and/or RF-positive RA, and not for seronegative RA [24]. Data from NHANES 2009–2012 showed that age was associated with the prevalence of gingival disease, and that 46% of dental adults in the US aged ≥30 years had gingival disease [23]. Other studies were consistent with these data and showed that the prevalence of gingival disease in RA patients was significantly associated with age [16, 32, 33]. A study evaluating the prevalence and severity of gingival disease in 40 RA patients showed that the prevalence of gingival disease was higher in men, smokers, and older patients [34, 35]. Interestingly, a recent report suggested that males had a strong inflammatory response to bacterial infection, and male neutrophils responded to lipopolysaccharide in vitro with increased chemokine expression, and increased osteoclast-driven bone-loss compared to females [36]. We found no association of age with gingival disease in the included population.

Previous results suggested that smoking is a trigger for ACPA-positive RA in genetically susceptible individuals [17]. Interestingly, the efficacy of DMARD treatment for RA was attenuated in smokers, and smoking cessation was beneficial for non-surgical periodontal therapy [15, 37, 38]. In our present study, there was a higher proportion of non-smokers among the no gingival disease group. Low physical activity levels were previously shown to correlate with increased odds of gingival disease [39, 40]. However, it was not clear if the risk of gingival disease was associated with physical activity in RA patients. Our data found no association of physical activity with gingival disease.

There are limited data describing the association between vitamin D levels and gingival disease. Vitamin D is thought to impact gingival disease via immunomodulatory or antimicrobial effects, calcium absorption, and effects on bone metabolism. High serum levels of 25 (OH) D substrate induce various immune cells to produce 1, 25 (OH)2 D which regulates the immune response at sites of inflammation, and inhibits monocyte production of cytokines such as IL-1β and TNFα which impair wound healing and induce bone resorption [41, 42]. It has been reported patients with periodontitis had significantly lower levels of vitamin D compared to controls [43]. Our data showing that the odds of gingival disease in RA patients decreased with an increase in serum vitamin D levels, are consistent with the previously reported role vitamin D in gingival disease [44].

Low vitamin C intake, and low serum ascorbic acid concentrations have previously been reported to be associated with an increased risk of gingival disease [45, 46], and this was attributed to the antioxidant and wound-healing activities of vitamin C. Indeed, vitamin C intake above the recommended daily allowance was shown to be beneficial in gingival disease treatment [47]. Our data were also consistent with these results, and showed that in RA patients, the odds of gingival disease were decreased with increased vitamin C intake.

The study of de Pablo et al (2008) also evaluated the association of periodontal disease with RA [48]. The study of de Pablo et al. differed from the current study reported here in that Pablo et al. used data from NHANES III (1988 to 1994) cycle and included patients ≥60 years of age. The study of de Pablo et al. included 4461 participants, 103 of whom had RA. They found that participants with RA had more missing teeth but less decay compared with non-RA participants. Multivariate analysis found the subjects with RA were more likely to be edentulous and have periodontitis. The findings of our study and that of de Pablo et al. support the association of gingival disease (periodontitis) with RA.

Goh et al. (2016) also used the NHANES III (1988 to 1994) database to assess if elevated serum IgG antibodies to 19 periodontal species were associated with the presence of rheumatoid factor (RF) in dentate participant ≥60 years of age who were not diagnosed with RA [49]. They found no significant association between antibodies to periodontal bacteria and being seropositive for RF. It is difficult to compare the findings of Goh et al. with ours and those of de Pablo et al.; the latter two studies investigated the relationship of gingival disease with clinically diagnosed RA. It is conceivable that factors associated with gingival disease differ between pre-clinical RA and clinical RA.

Our present study has expanded our understanding of the association between gingival disease and RA, in addition to defining important protective factors of gingival disease using the NHANES database. The major limitation of this study was that it was a cross-sectional study, and therefore the results cannot be implied to determine cause and effect. Additionally, the data were sourced from answers to questionnaire given to the general public, and not from medical records. There could therefore have existed recall bias. Only NHANES III (1988–1994) has blood measurements on rheumatoid factors, the information from the other NHANES cycles was based on questionnaire of history or RA. Since the current study included data from NHANES 1999–2004, the diagnosis of RA was based on self-report. The use of NHANES 1999–2004 was chosen as these cycled captured the relevant periodontal and RA information. However, the design of the NHANES questionnaire is such that the self-report has a certain degree of discrimination and reliability. The respondent must answer yes or no to if a doctor or healthcare professional has told them they have arthritis, and must indicate the age of diagnosis and the type of arthritis. Only subjects who could respond to these questions were included in the current study. In addition, our study did not include patients >85 years of age, due to the fact the NHANES database only records if a person is over the age of 85, and not a specific age. Given the typical age profile of RA, excluding this age group may have confounded our findings. In addition, the periodontal examination methodology used for part of the time frame included in the current study differed (from partial to full mouth periodontal examination) which may have resulted in a significant underestimate of the prevalence of periodontal disease within the included study population. The duration of RA reported in the NHANES database is limited by the fact the duration was calculated as age at NHANES screening minus age at RA diagnosis. It is possible a participant had RA prior to diagnosis. Given the extensive evidence suggesting a significant association between gingival disease and RA, it will be important to validate our data in prospective, population-based cohort studies and define in greater detail the major risk factors of gingival disease. It will also be interesting to use data sourced from medical records to validate our current findings.

### Conclusions

Our present study evaluated risk factors of gingival disease in 754 RA patients with gingival disease. Our data showed that the risk of gingival disease was significantly higher among male RA patients, and was significantly lower with increased vitamin C intake and increased serum levels of vitamin D. Given the significant association between the prevalence of gingival disease and RA, identification of risk factors of gingival disease will be useful as a screening tool in national health surveys to improve the management of periodontal disease as well as RA.
